# Challenges of recruiting elderly patients undergoing cardiac surgery - our experience of recruiting 2500 participants in just 30 months at a single centre

**DOI:** 10.1186/1745-6215-14-S1-P87

**Published:** 2013-11-29

**Authors:** Zoe Plummer, Emma Hopkins, Lucy Culliford, Chris Rogers, Barnaby Reeves, Gavin Murphy, Veerle Verheyden, Andrew Mumford

**Affiliations:** 1 University of Bristol, Bristol, UK; 2 University Hospitals Bristol NHS Foundation Trust, Bristol, UK; 3 University of Leicester, Bristol, UK

## Background

Emergency operations, cancellations, changing operating theatre schedules, day of surgery arrivals and an aging population are all challenges facing the recruitment of cardiac surgery patients into studies. The observational Coagulation and Platelet Function Testing in Cardiac Surgery (COPTIC) study, which required patients to consent to give blood samples at the start and end of surgery, is the largest of its kind and the largest single study conducted at our hospital. We describe our experience of recruitment and retention and share our successful strategy.

## Methods

Adults with capacity having cardiac surgery were eligible to participate. Information sheets were posted to elective patients waiting at home, faxed to patients waiting in in other hospitals and handed to those arriving at hospital at short notice. Waiting list coordinators and ward staff notified the team of new arrivals and up to 8 potential participants were screened, approached and consented each day. To minimise the number of patients missed, a team member was available 12 hours/day Monday to Friday and Sunday evenings. Emergency patients were able to participate on the basis of prospective verbal consent given to their surgeon. Written consent was obtained from these patients after their operation.

## Results

During a 30-month recruitment period 3056 patients were approached and 2530 consented. Blood samples were collected for >95% of participants. One patient withdrew consent.

## Conclusions

This study recruited above target and was delivered ahead of time. Recruitment is a process, requiring a period of evolution and a team committed to the common aim.

## Disclaimer

This abstract presents independent research funded by the National Institute for Health Research (NIHR under its Programme Grants for Applied Research Programme(*Grant Reference Number RP-PG-0407-10384*). The views expressed are those of the author(s) and not necessarily those of the NHS, the NIHR or the Department of Health.

